# Chronic corticosterone administration induces negative valence and impairs positive valence behaviors in mice

**DOI:** 10.1038/s41398-019-0674-4

**Published:** 2019-12-10

**Authors:** Andrew Dieterich, Prachi Srivastava, Aitesam Sharif, Karina Stech, Joseph Floeder, Samantha E. Yohn, Benjamin A. Samuels

**Affiliations:** 1 Neuroscience Graduate Program, Rutgers, The State University of New Jersey, Piscataway, NJ 08854 USA; 2 Department of Psychology, Behavioral and Systems Neuroscience Area, Rutgers, The State University of New Jersey, 152 Frelinghuysen Rd, Piscataway, NJ 08854 USA; 3 Department of Pharmacology, Vanderbilt University, Nashville, TN USA

**Keywords:** Psychology, Neuroscience, Psychology, Neuroscience

## Abstract

Behavioral approaches utilizing rodents to study mood disorders have focused primarily on negative valence behaviors associated with potential threat (anxiety-related behaviors). However, for disorders such as depression, positive valence behaviors that assess reward processing may be more translationally valid and predictive of antidepressant treatment outcome. Chronic corticosterone (CORT) administration is a well-validated pharmacological stressor that increases avoidance in negative valence behaviors associated with anxiety^1–4^. However, whether chronic stress paradigms such as CORT administration also lead to deficits in positive valence behaviors remains unclear. We treated male C57BL/6J mice with chronic CORT and assessed both negative and positive valence behaviors. We found that CORT induced avoidance in the open field and NSF. Interestingly, CORT also impaired instrumental acquisition, reduced sensitivity to a devalued outcome, reduced breakpoint in progressive ratio, and impaired performance in probabilistic reversal learning. Taken together, these results demonstrate that chronic CORT administration at the same dosage both induces avoidance in negative valence behaviors associated with anxiety and impairs positive valence behaviors associated with reward processing. These data suggest that CORT administration is a useful experimental system for preclinical approaches to studying stress-induced mood disorders.

## Introduction

Mood disorders are a major burden on society and are extremely prevalent. An estimated 21.4% of adults in the United States experience a mood disorder at some point in their lives^5^ and ~16.2 million adults experience a major depressive episode each year^6^. Specific symptoms of major depressive disorder must include depressed mood and/or anhedonia^7^. Anhedonia is the lack of feelings of pleasure and the positive experience from reward^8^. Therefore, depression can include both a gain of negative affect and/or a loss of positive affect. Most preclinical rodent research focuses on assessing anxiety-associated behaviors that are consistent with a gain of negative affect. However, instrumental behaviors associated with positive valence in rodents may have more translational value than anxiety-related approach-avoidance behaviors associated with negative valence because relatively similar instrumental behaviors can be performed in rodents and humans to study reward learning^9^, outcome devaluation^10^, progressive ratio^11^, and probabilistic reversal learning tasks^12,13^. This conservation across species suggests that increased adoption of using positive valence behaviors in rodents might significantly accelerate therapeutic development.

Chronic stress can precipitate mood disorders and several distinct chronic stress paradigms are widely used to study how stress impacts anxiety-associated approach-avoidance negative valence behaviors and neural circuitry in rodents. However, relatively few studies have assessed whether these chronic stress paradigms also impact positive valence behaviors including instrumental behaviors. Chronic corticosterone (CORT) administration is one such paradigm that can mimic the effects of stress on anxiety-associated approach-avoidance negative valence behaviors^1^. CORT is the rodent stress hormone analog of cortisol in humans and is the major output of the hypothalamus-pituitary-adrenal (HPA) axis, which is hyperactive in humans with major depressive disorder and in rodents exposed to chronic stress^14^. Specifically, chronic CORT administration induces an anxiety-associated negative valence phenotype, characterized by increased avoidance of the center in the open field, increased avoidance of the open arms in the elevated plus maze, and increased latency to eat in the novelty suppressed feeding test^1^. Taken together, these increased avoidance behaviors suggest that chronic CORT administration can induce an anxiety-related negative affective state.

Here we show that chronic CORT administration not only induces avoidance in anxiety-related negative valence behaviors, but also impairs positive valence instrumental behaviors. Following instrumental training, we utilized several instrumental behavioral tests, including outcome devaluation, progressive ratio, and probabilistic reversal learning, to assess the effects of chronic CORT on components of reward processing. Overall, CORT administration impaired instrumental acquisition, reduced sensitivity to a devalued outcome, reduced breakpoint in progressive ratio, and impaired performance in probabilistic reversal learning. Taken together, these data suggest that chronic stress paradigms, such as CORT administration, that are already validated with anxiety-associated approach-avoidance negative valence behaviors may also be useful for studying translationally relevant instrumental behaviors associated with positive valence.

## Materials and methods

### Mice and drug treatment

All experiments were conducted in compliance with NIH laboratory animal care guidelines and approved by Rutgers University Institutional Animal Care and Use Committee. All mice were maintained on a 12L:12D schedule where the lights were on from 6:00 a.m. to 6:00 p.m., and run in behavioral tests between 9:00 a.m. and 2:00 p.m. daily. Adult male C57BL/6 mice (age 7 weeks) (Jackson Labs, Bar Harbor, ME) were randomly divided into Vehicle and Corticosterone (CORT) treatments. Vehicle-treated mice (*n* = 20) received beta-cyclodextrin dissolved in their drinking water, while CORT-treated mice (*n* = 30) received CORT (35 μg/mL) (Sigma-Aldrich, St. Louis, MO) and beta-cyclodextrin (4.5 mg/mL) (Sigma-Aldrich, St. Louis, MO) dissolved in their drinking water (5 mg/kg/day CORT). Sample sizes were estimated to achieve adequate power. After 4 weeks of this treatment, mice were food-restricted and maintained at 85–93% of their free-feeding body weight. To ensure the mice maintained this body weight they were weighed daily and given standard lab chow at least 1 h after completing testing each day. Training conditions were collapsed into Vehicle (*n* = 19) and CORT (*n* = 26) treatment, as after initial instrumental acquisition there were no differences between training conditions on behavior. A total of 5 mice (*n* = 1 Vehicle; *n* = 4 CORT) died or did not reach criterion for acquisition, and were excluded from all further analyses. A smaller, separate cohort (*n* = 10 for both Vehicle and CORT treatments) of adult male C57BL/6 mice was used for limited-training outcome devaluation and contingency degradation tests. In the negative valence cohort (*n* = 20 for both Vehicle and CORT treatments), adult male C57BL/6 mice were tested in the open field (OF), novelty-suppressed feeding (NSF), and forced swim (FST) tests after 4 weeks of CORT or Vehicle (5 mg/kg/day) administration. All mice were run in the operant tests by individuals blind to the details of the experiment, between 9:00 a.m. and 4:00 p.m. daily.

### Open field

Vehicle (*n* = 20) and CORT (*n* = 20) mice were placed in a corner of Plexiglas open field (OF) chambers measuring 43 × 43 cm, and Motor Monitor software (Kinder Scientific, Poway, CA) was used to measure distance traveled (cm), and both entries and time in the center of the OF, detected with infrared photobeams on the walls, and the center defined as a square 11 cm from each wall of the OF.

### Novelty-suppressed feeding

Vehicle (*n* = 10) and CORT (*n* = 10) mice were food-deprived 18 h before novelty-suppressed feeding (NSF) testing. Mice were placed in the corner of a novel, brightly-lit NSF chamber with a single food pellet placed in the center, for a 6-min test. The NSF is a test assessing the conflict between motivation to eat while in a food-deprived state, and the aversive nature of the novel, brightly-lit arena. Latency to approach the pellet and take a bite was recorded.

### Forced swim

Vehicle (*n* = 20) and CORT (*n* = 20) mice were placed in forced swim (FST) chamber containing room temperature water for 6 min, and Motor Monitor software (Kinder Scientific, Loway, CA) was used to assess immobility, measured as 6 or less beam breaks in 5 s, during the final 4 min of the test.

### Operant chambers

A large, separate cohort of Vehicle (*n* = 20) and CORT (*n* = 30), and a second, smaller cohort of Vehicle (*n* = 10) and CORT (*n* = 10) mice were trained and tested in standard mouse operant chambers (Med Associates, Fairfax, VT) housed in sound-attenuating cubicles, in a designated behavioral testing room. The operant chambers were coupled to a power control and interface connected to a computer running the Med-PC IV software (Med Associates, Fairfax, VT). The operant chambers contained two retractable response levers on one wall, and two 20 mg food pellet hoppers attached by Y-tubing to a single delivery port between the levers. Grain-based food pellet reinforcers (Bio-Serv, Flemington, NJ) were delivered from each hopper into the delivery port for the mouse to consume.

### Operant conditioning

Mice were magazine-trained with pellets automatically delivered every 30–60 s for a single session. After 2 days, no pellets were automatically dispensed, and the mice were conditioned to lever press on a continuous reinforcement (CRF) schedule until they made 30 responses on both levers in consecutive sessions (criterion ≥30 lever presses on both response levers in consecutive sessions).

### Extended-training outcome devaluation

The larger cohort of mice (*n* = 19 Vehicle; *n* = 26 CORT) first completed extended-training, satiety-specific outcome devaluation testing to determine if chronic CORT affects responding for a devalued outcome. One hopper, counterbalanced across mice, was filled with chocolate-flavored (Bio-Serv) pellets, while the other continued to deliver standard grain-based food pellets. Thus, responding on one of the two levers led to delivery of a chocolate-flavored pellet, and responding on the other led to delivery of a grain-based pellet. Mice were subjected to two sessions of each of the following in succession: CRF, Random Ratio 5 (RR5) Schedule, RR10, and RR20. For each random ratio schedule, 5, 10, or 20 lever presses, on average, were required for reinforcer delivery, respectively. This protocol of extended-training has been used previously (Dias-Ferreira et al., 2009). After RR20 training, the mice progressed to satiety-specific devaluation of either the grain or chocolate pellets, as they were given free access to one of the pellet types in a fresh cage for one hour. Immediately after this they were given a 5-min extinction test and lever presses were recorded on both levers. If the mouse received free access to the grain pellets, the lever associated with the grain pellets was considered the devalued condition, and the lever associated with the chocolate pellets was considered the valued condition, and vice versa. Lever presses on both sides were quantified.

### Extended-training contingency degradation

Mice were then re-trained on a RR20 schedule on both levers until responding stabilized, still for both grain-based and chocolate-flavored pellets, counterbalanced by side. They then continued on one active lever on the RR20 schedule, while the second lever became inactivated, as pellets were delivered based on the rate of responding on the active lever, to degrade responding for that outcome. When responding on the degraded lever had robustly decreased in both Vehicle and CORT mice, a 5-min extinction test session was conducted, to determine if chronic CORT impacts responding for a degraded contingency.

### Limited-training outcome devaluation

As over-training causes a shift to habitual control of responding, in the limited-training outcome devaluation test a separate cohort of Vehicle (*n* = 10) and CORT (*n* = 10) mice was briefly habituated to and trained on a CRF schedule to lever press on one response lever for the grain-based food pellets. Mice were then trained on a variable ratio 2 (VR2) schedule of reinforcement for three days, where 1, 2, or 3 responses led to reinforcer delivery, after which they were given free access to either the reinforcer pellets (devalued condition), or standard lab chow (valued condition) for one hour prior to a 5-min extinction test. Mice completed both conditions in separate sessions, with 2 days in between testing to test if CORT affects sensitivity to a devalued outcome after limited instrumental training.

### Limited training contingency degradation

For contingency degradation, Vehicle (*n* = 10) and CORT (*n* = 10) mice from the limited-training outcome devaluation experiment were re-trained on a VR2 schedule but now on both response levers, for 3 days, or until responding stabilized. Then, one of the levers was retracted and responding on the other lever (non-degraded condition) was recorded in one VR2 session, counterbalanced across mice, as described previously^15,16^. In the next session the other lever was ejected into the chamber (degraded condition) and pellets were dispensed at the same rate as in the previous day’s VR2 session, to degrade responding on the degraded lever. A 5-min extinction test session was conducted the following day to assess if CORT reduces sensitivity to a degraded contingency.

### Progressive ratio

After completing extended-training contingency degradation, the cohort of Vehicle (*n* = 19) and CORT (*n* = 26) mice completed three separate progressive ratio test sessions, to test for motivation to expend effort. In each, the requirement to obtain a food pellet reinforcer increased linearly (1, 5, 9, X + 4) until the mice ceased to respond for 5 min, or until 4 h, and was recorded as the breakpoint, which provides a measure of motivation to work to obtain a reinforcer. Total active lever presses were also measured in each session. After completing all three progressive ratio tests, the large cohort progressed to reversal learning.

### Reversal learning

Vehicle (*n* = 19) and CORT (*n* = 26) mice were then trained in 30-min sessions on a reversal learning procedure, where they were reinforced on a CRF schedule for each response made on either the left or right lever (correct), counterbalanced between mice, while the other lever (incorrect) was not reinforced. After eight consecutive responses, recorded as a completed reversal on the correct lever, the correct and incorrect levers switched, and the previously incorrect, non-reinforced lever was now the correct, reinforced lever. With an incorrect response, the counter started over and the mouse was required to make an additional eight consecutive correct response to complete the reversal and switch the contingencies. Correct and incorrect levers switched with each completed reversal in each reversal learning session. Correct responses, omissions (no response made within 10 s), incorrect responses, and completed reversals were measured. Following five sessions of reversal learning, the mice completed three different probabilistic reversal learning (PRL) test sessions, which were each separated by 2 days of reversal learning sessions.

### Probabilistic reversal learning

PRL was conducted as previously described^17,18^ in Vehicle (*n* = 19) and CORT (*n* = 26) mice. The procedure for PRL is similar to reversal learning, except PRL can be divided in to three separate sessions involving different reinforcement probabilities. In each, the correct lever is reinforced on only 70, 80, or 90% of correct presses, while the incorrect lever is reinforced on 10, 20, or 30% of the incorrect presses, respectively, which is referred to as pPCR, or the probability of punishment for a correct response. Therefore, the three PRL sessions are referred to as 0.1, 0.2, and 0.3 pPCR. These probabilities of reinforcement induce misleading positive and negative feedback. A lose-shift occurred when a mouse switches to respond on the incorrect lever immediately after making a correct, but not reinforced response. A win-stay occurs when a mouse continues to respond on the correct lever after a correct response made in the immediately preceding trial. These are measures of negative and positive feedback, respectively, and were assessed along with completed reversals for each of the three PRL test sessions.

### Fear conditioning

A separate cohort of Vehicle (*n* = 17) and CORT-administered (*n* = 18) mice were habituated to an operant chamber for 180 s, followed by a 2-s 0.7 mA foot-shock through the grid floor of the operant chamber, followed by an additional 15 s prior to removing the mice. In total, 24 h later, the mice were placed in the same context for an 180-s fear retrieval session, and video was recorded by digital cameras above the chambers, in order to test contextual fear conditioning. Percent time freezing was quantified in both training and test sessions by a blind observer as the absence of movement besides respiration.

### Experimental design and statistical analysis

The effect of chronic CORT treatment on behavior was assessed using separate one-way ANOVAs, *t*-tests, two-way repeated measures ANOVAs, or Kaplan–Meier Survival analysis (NSF). Separate cohorts of Vehicle and CORT-treated mice were used for separate experiments. Vehicle (*n* = 20) and CORT (*n* = 20) mice were used for open field and forced swim tests, and Vehicle (*n* = 10) and CORT (*n* = 10) for NSF. Vehicle (*n* = 10) and CORT (*n* = 10) mice were used in the limited-training outcome devaluation cohort. Vehicle (*n* = 19) and CORT (*n* = 26) mice were used for the extended training outcome devaluation, tested for sessions to acquisition, progressive ratio, and probabilistic reversal learning tests. Planned multiple comparisons were made to determine significance between groups.

## Results

### Negative valence

**Fig. 1:**
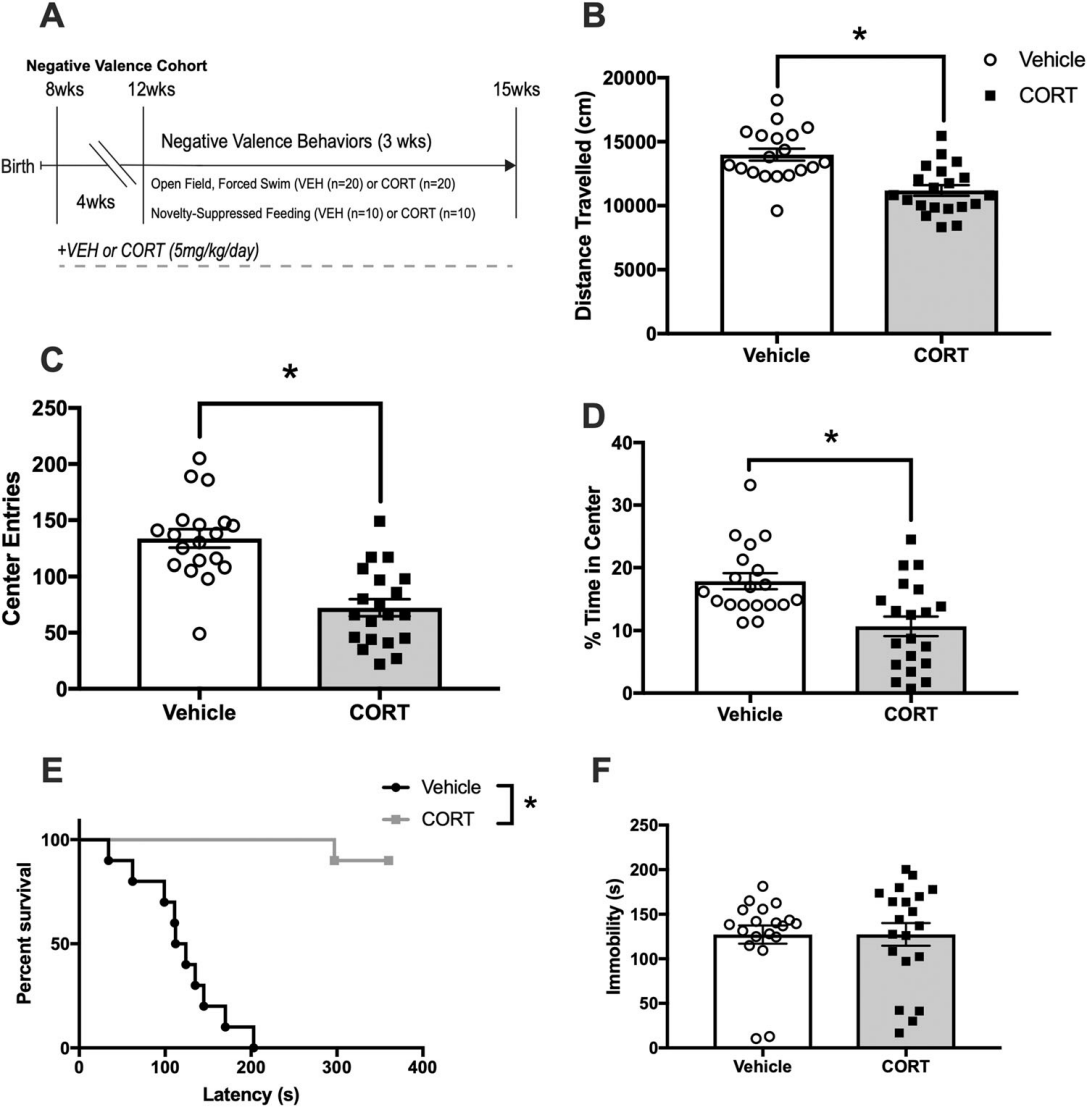
Chronic CORT increases negative valence. **a** Vehicle (*n* = 20) or CORT (*n* = 20) C57BL/6 mice were administered CORT or Vehicle (5 mg/kg/day) in their drinking water for 4 weeks prior to negative valence behaviors, including the open field (OF), novelty-suppressed feeding (NSF), and forced swim (FST) tests. **b** In the OF, CORT reduced locomotor activity, measured as distance traveled (cm) in a 30-min session (*t*(37) = 4.5, *p* *<* 0.001). **c** CORT also reduced center entries in the OF, (*t*(37) = 5.5, *p* *<* 0.001), **d** as well as percent time in the center (*t*(37) = 3.5, *p* *<* 0.001), both measures of avoidance behavior in the OF. **e** In the NSF assay, chronic CORT increased latency to take a bite of a food pellet in the center of the brightly-lit arena under a food-deprived state (Kaplan–Meier survival, *X*^2^(2, *N* = 36) = 16, *p* *<* 0.001). **f** In the FST, CORT had no effect on immobility, indicative of behavioral despair (*t*(37) = 0.017), *p* *=* 0.987), a commonly used depressive test to assess antidepressant response. Values plotted are mean ± SEM. *p *<* 0.001. Taken together, chronic CORT induces a robust negative valence behavioral phenotype characterized by increased avoidance in the OF and NSF, while not increasing immobility in the FST.

To confirm that chronic CORT administration (5 mg/kg/day) mimics the effects of chronic stress, we assessed behavior in the open field (OF) and forced swim test (FST) (Fig. 1a) in Vehicle (*n* = 20) and CORT-administered (*n* = 20) mice, as well as novelty-suppressed feeding (NSF) in a separate group of Vehicle (*n* = 10) and CORT-administered (*n* = 10) mice. Similar to previous reports^1^, unpaired *t*-tests revealed that chronic CORT administration reduced overall locomotor activity in the OF (*t*(37) = 4.5, *p* *<* 0.001, Fig. 1b), entries into the center of the OF (*t*(37) = 5.5, *p* *<* 0.001, Fig. 1c), and percent time spent in the center (*t*(37) = 3.5, *p* *<* 0.001, Fig. 1d). Furthermore, chronic CORT increased latency to eat in NSF (Kaplan-Meier survival, X2(2, *N* = 9) = 22, *p* < 0.0001) (Fig. 1e) in a separate cohort of Vehicle (*n* = 10) and CORT-administered (*n* = 10) mice, without affecting latency to eat in the home cage (*t*(18) = 0.83), *p* = 0.419) (Supplemental Fig. 1a), suggesting the increase in latency is not affected by group differences in hunger. However, similar to previous reports^1^, chronic CORT did not affect immobility in antidepressant-sensitive FST (*t*(37) = 0.017), *p* *=* 0.987, Fig. 1f). Taken together, these results confirm that chronic CORT administration induces negative valence behaviors associated with potential threat in the OF and NSF.

### Instrumental acquisition

**Fig. 2:**
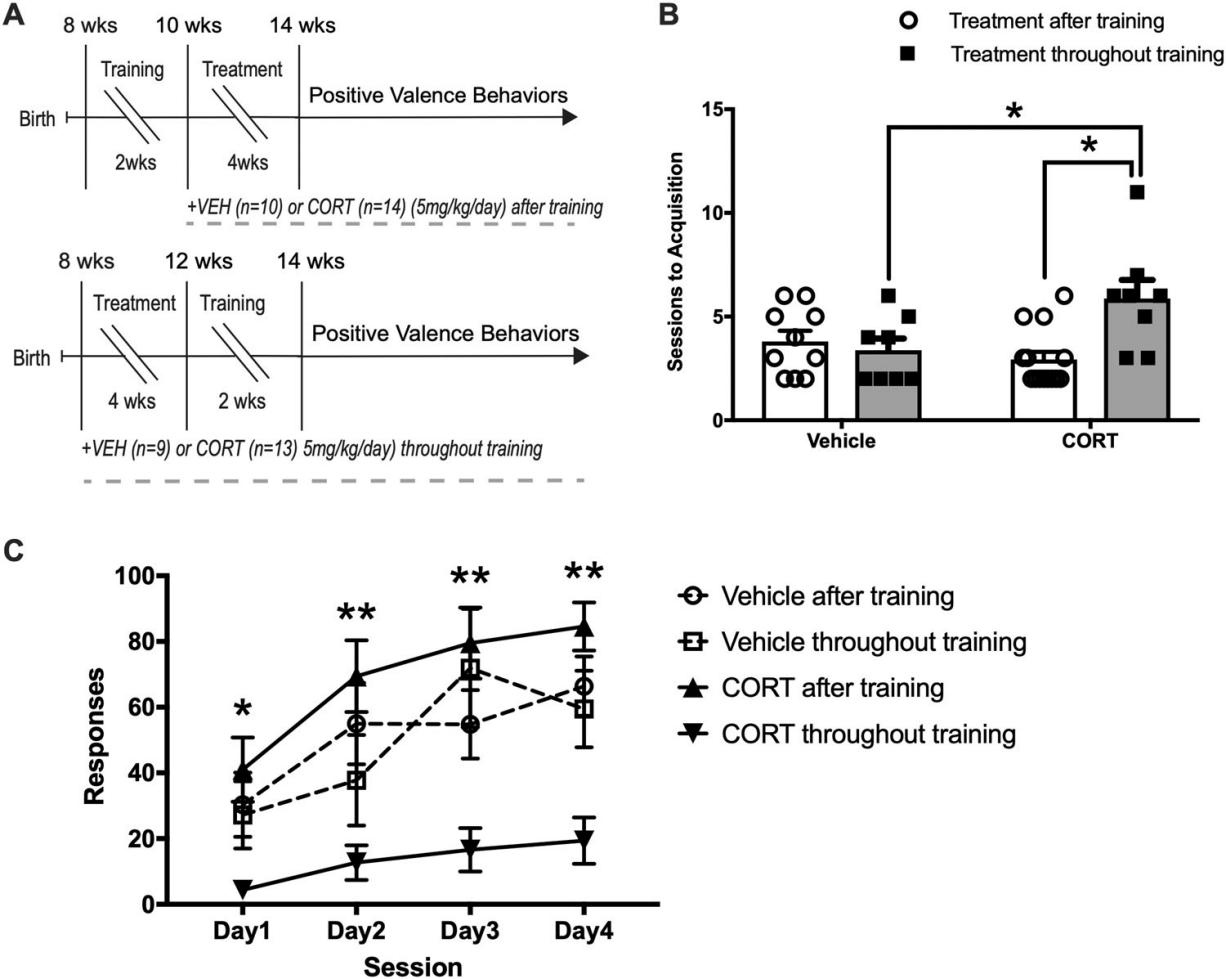
Chronic CORT impairs instrumental acquisition. **a** Vehicle (*n* = 10, both training conditions) and CORT (*n* = 15, both training conditions) mice were administered CORT or Vehicle (5 mg/kg/day) in their drinking water for 4 weeks prior to instrumental acquisition (Vehicle or CORT throughout training), or after instrumental acquisition (Vehicle or CORT after training). **b** Sessions to acquisition (criterion defined as consecutive sessions with ≥30 lever presses on both response levers) was assessed in Vehicle and CORT mice throughout or after treatment. CORT throughout training mice required more acquisition sessions to reach criterion compared to CORT after training (*p* *=* 0.003), and Vehicle throughout training (*p* *=* 0.035). **c** Responses were recorded throughout acquisition and were reduced by CORT treatment throughout training compared to CORT treatment after training (Day 1: *p* *=* 0.023, Days 2, 3, and 4: *p* *<* 0.001) and compared to Vehicle treatment throughout training on Days 3 (*p* *<* 0.001) and 4 (*p* *=* 0.029). Values plotted are mean ± SEM. *p *<* 0.05, **p *<* 0.001. Taken together, CORT throughout training inhibits instrumental acquisition, reducing lever presses during acquisition, and increasing sessions to reach criterion for acquisition.

We first assessed the effects of CORT on instrumental acquisition using a distinct cohort of mice than the negative valence behaviors. We tried two different protocols, where CORT was either administered throughout training or after instrumental training (Fig. 2a). A two-way ANOVA revealed a main effect of training on sessions to acquisition (*F*(1, 36) = 4.88, *p* *=* 0.034) and an interaction between training and CORT administration (*F*(1,36) = 8.726, *p* *=* 0.006) (Fig. 2b). Multiple comparisons revealed that CORT administration throughout training resulted in significantly more sessions to acquisition than mice that were administered CORT after the completion of training (*p* *=* 0.003) or were administered vehicle throughout training (*p* *=* 0.035). We also assessed total number of lever press responses made across the first four training sessions. A two-way repeated measures ANOVA revealed a main effect of day on responses during training (*F*(3, 126) = 28.36, *p* *<* 0.001), a main effect of CORT administration, (*F*(3, 42) = 8.50, *p* *<* 0.001), and a significant day × CORT administration interaction, (*F*(9, 126) = 2.30, *p* *=* 0.02) (Fig. 2c). Mice that received CORT throughout training made significantly less responses than mice that received CORT after the completion of training on all 4 training days (Day 1: *p* *=* 0.023, Days 2, 3, and 4: *p* *<* 0.001) and mice that received vehicle throughout training on Days 3 (*p* *<* 0.001) and 4 (*p* *=* 0.029). Next, we assessed learning index for all groups, calculated as correct lever responses divided by total number of trials, to give a per trial index of learning across instrumental acquisition. A one-way ANOVA for learning index in session 6 revealed a significant difference between groups (*F*(3, 35) = 4.09, *p* = 0.0137) (Supplemental Fig. 2a). Planned comparisons indicate CORT throughout training reduces the learning index in session 6 compared to CORT after training (*p* = 0.043) (Supplemental Fig. 2b). However, CORT throughout training and Vehicle throughout training had a similar learning index (*p* = 0.236). Thus, Vehicle and CORT-administered mice receiving treatment throughout training reach a similar learning index prior to advancing to further operant behavioral testing. Further, contextual fear conditioning was assessed in a separate group of Vehicle (*n* = 17) and CORT-administered (*n* = 18) mice. A repeated-measures two-way ANOVA revealed a main effect of session (training or test) (*F*(1, 31) = 1116, *p* *<* 0.001), but no main effect of CORT administration (*F*(1, 31) = 0.09, *p* = 0.767), and no interaction (**F**(1, 31) = 0.084, p = 0.774) on percent time freezing during contextual fear conditioning and retrieval sessions (Supplemental Fig. 2c). This provides additional evidence chronic CORT does not impair associative learning. Taken together, these data demonstrate that CORT administration throughout training impairs instrumental acquisition compared to Vehicle and to CORT administration after training.

### Extended-training devaluation and degradation

**Fig. 3:**
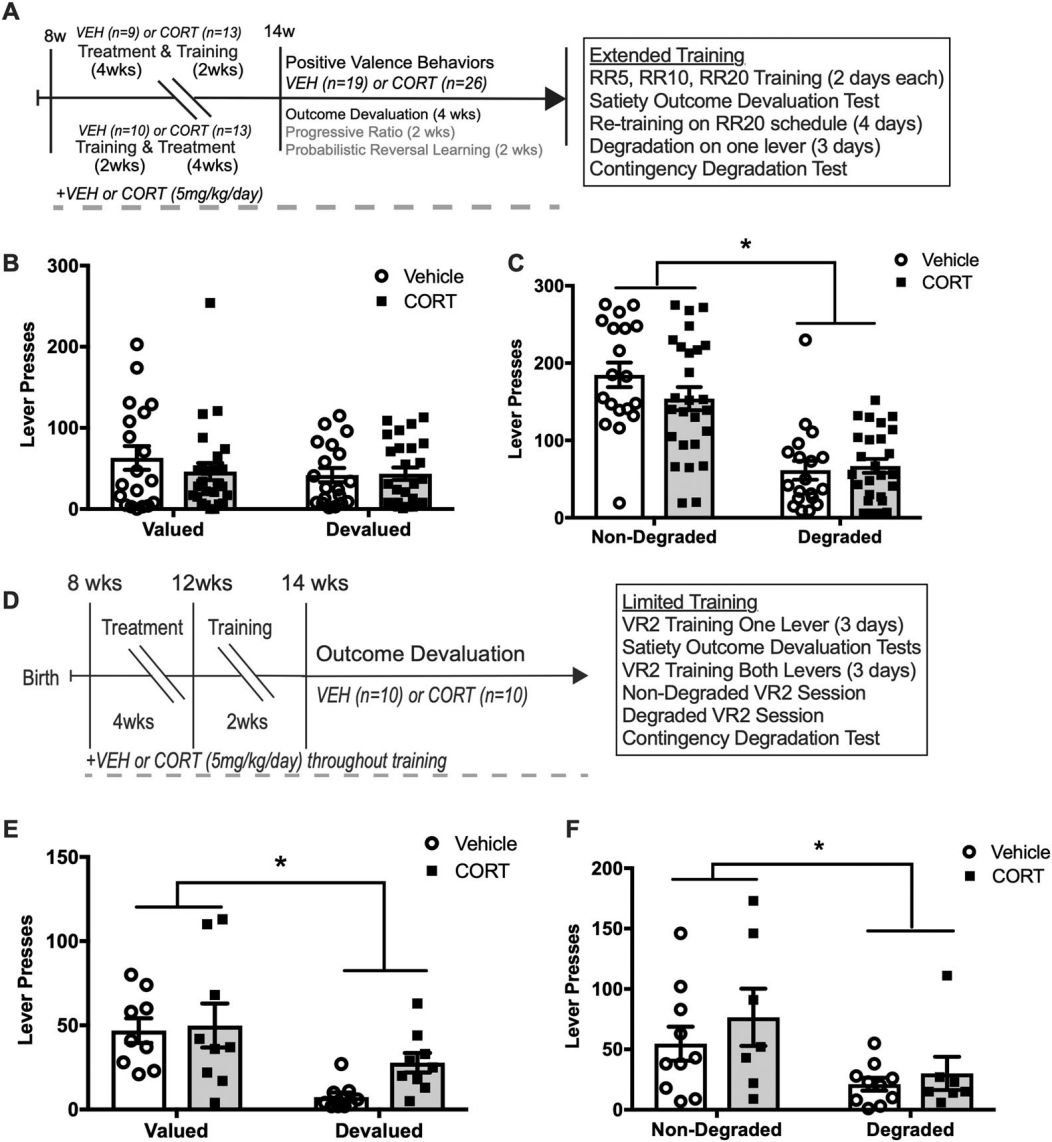
Chronic CORT impairs outcome devaluation after limited instrumental conditioning, but not extended training. **a** Training groups (treatment after and treatment throughout training) were collapsed into treatments (Vehicle and CORT) after initial instrumental acquisition. Mice were tested for satiety-specific outcome devaluation and contingency degradation after both extended and limited-training, to test for the effect of chronic CORT on sensitivity to a devalued or degraded outcome, and whether goal-directed or habitual mechanisms are guiding behavior. **b** Vehicle (*n* = 19) and CORT (*n* = 26) mice did not differ in lever pressing in extinction test sessions after satiety-specific outcome devaluation (*p* *>* 0.05). **c** Vehicle (*n* = 19) and CORT (*n* = 26) mice similarly reduced lever pressing on a degraded response lever (*p* *<* 0.001 for both treatments), but not on a non-degraded lever (*p* *>* 0.05), suggesting extended-training similarly degraded responding on one lever in both treatments, as the lever was degraded for 3 degradation sessions**. d** Next, a separate cohort of Vehicle (*n* = 10) and CORT (*n* = 10) mice were trained in a shorter protocol before satiety-specific outcome devaluation and contingency degradation, to examine if the extended-training protocol led to over-training. **e** After 1-h free access to reinforcer pellets (devalued), or standard lab chow (valued), limited-training led to a reduction in lever pressing in the devalued condition in Vehicle mice (*p* *=* 0.007) but not in CORT mice (*p* *=* 0.144), suggesting CORT reduces sensitivity to a devalued outcome after limited training. **f** However, limited training did not lead to reduced responding for a degraded outcome like shown with extended-training in either Vehicle (*p* *=* 0.247), or CORT (*p* *=* 0.156) mice. Values plotted are mean ± SEM. *p *<* 0.05, **p *<* 0.001. Taken together, chronic CORT reduces sensitivity to a devalued outcome after limited trained, without influencing contingency degradation, while extended-training shifts behavior to habitual responding in both treatments.

Since all mice reached the acquisition criterion, we collapsed training groups for the remainder of behavioral testing. We next assessed whether continued CORT administration affected extended-training satiety-specific outcome devaluation in mice that completed instrumental acquisition (Fig. 3a). For satiety-specific outcome devaluation, a two-way repeated measures ANOVA for lever pressing revealed no main effect of CORT (*F*(1, 43) = 0.4, *p* *=* 0.529), value condition (*F*(1, 43) = 1.8, *p* = 0.186), or interaction (*F*(1, 43) = 1.1, *p* *=* 0.300) (Fig. 3b). Thus, Vehicle and CORT mice responded similarly on valued and devalued levers during the extinction test. This indicates that CORT does not impact extended-training outcome devaluation. Mice were next tested in extended-training contingency degradation, to determine if chronic CORT affects responding on a degraded contingency. For contingency degradation, a two-way repeated measures ANOVA for lever pressing revealed a main effect of degradation condition (*F*(1, 43) = 48, *p* *<* 0.001), but no effect of CORT, (*F*(1, 43) = 1.3, *p* *=* 0.267), and no interaction (*F*(1, 43) = 1.4, *p* *=* 0.238 (Fig. 3c)). This indicates both Vehicle and CORT mice reduce responding on a degraded lever compared to the non-degraded lever, suggesting this protocol produced a robust degradation effect in both treatments. Taken together, Vehicle and CORT mice both reduced responding for a reinforcer devalued by contingency degradation, suggesting that like outcome devaluation, CORT does not affect responding when compared to Vehicle.

### Limited-training devaluation and degradation

Since overtraining may have caused a shift to habitual control of responding in the extended-training condition, we also tried a limited-training protocol in a distinct cohort of mice (Fig. 3d). For limited-training outcome devaluation, a two-way repeated measures ANOVA revealed a main effect of value condition on responding during the extinction sessions after a 1-h period of free access to the reinforcer pellets (*F*(1, 16) = 14, *p* *=* 0.002), but a non-significant main effect of CORT administration (*F*(1, 16) = 2, *p* *=* 0.173), or interaction (*F*(1, 16) = 1.1, *p* *=* 0.302) (Fig. 3e). Planned comparisons indicated that the Vehicle mice show a significant devaluation effect (*p* *<* 0.007), while CORT mice did not show this effect (*p* *=* 0.144). Thus, chronic CORT administration impairs outcome devaluation, as CORT mice are insensitive to the devalued outcome. In a separate cohort of Vehicle (*n* = 10) and CORT-administered (*n* = 10) mice, chronic CORT did not affect reinforcer pellet consumption in the 1-h free access test (*t*(18) = 0.21, *p* = 0.84) (Supplemental Fig. 1b), suggesting the effect seen in outcome devaluation is due to habitual responding and not a difference in pellet consumption between Vehicle and CORT-administered mice. These mice were next assessed in a limited-training contingency degradation test. A two-way repeated measures ANOVA for the effect of contingency degradation on responding in an extinction session revealed a main effect of degradation condition (*F*(1, 15) = 6.2, *p* *=* 0.025), but no effect of CORT administration (*F*(1, 15 = 1.4, *p* *=* 0.248), or interaction (*F*(1, 15) = 0.16, *p* *=* 0.692) (Fig. 3f). Thus, Vehicle and CORT-administered mice did not show a degradation effect. Overall, these data demonstrate that chronic CORT reduces sensitivity to a devalued outcome when mice are briefly trained, while not affecting a degraded contingency between response and reinforcer.

### Progressive ratio

**Fig. 4:**
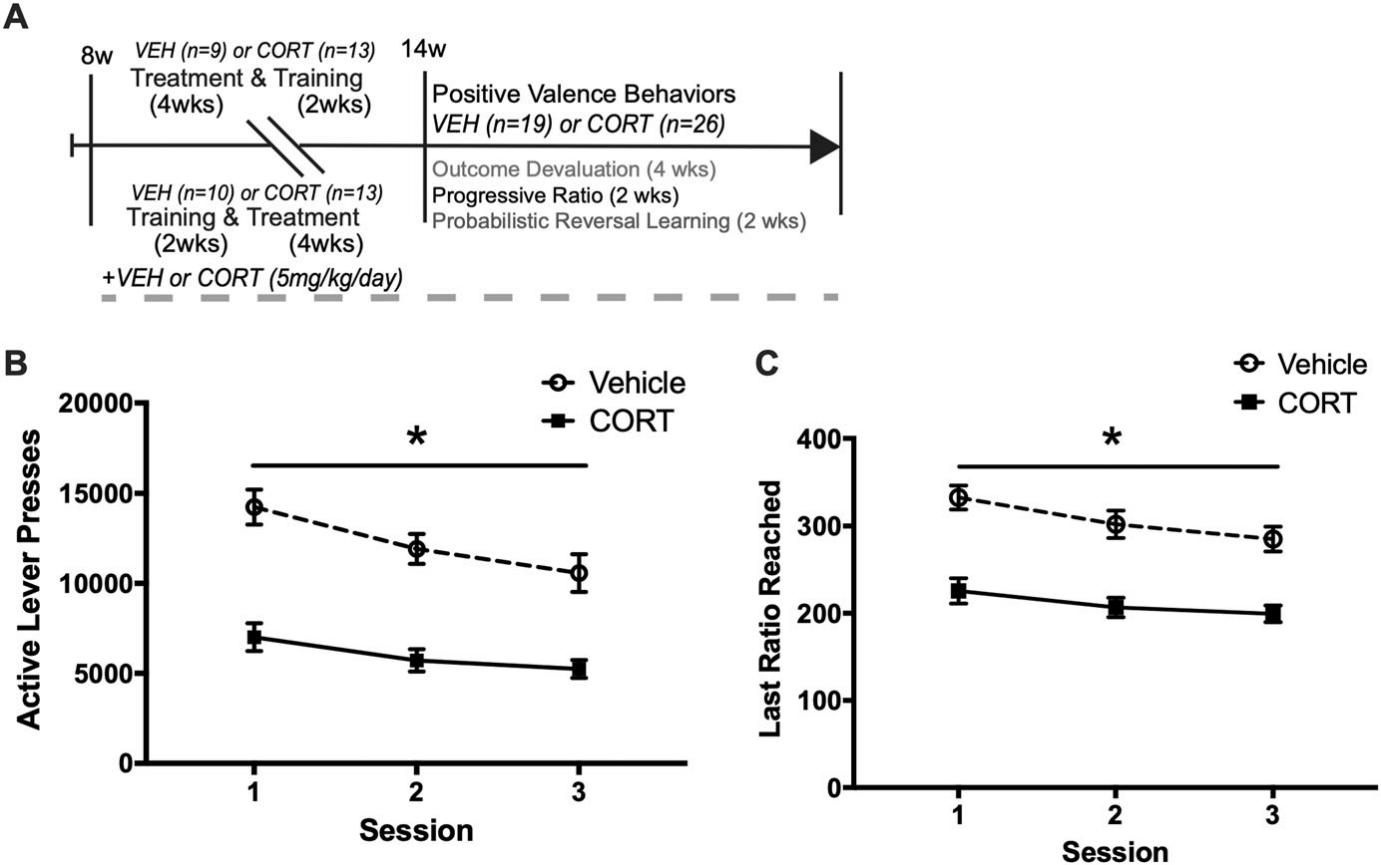
Chronic CORT reduces active lever presses and breakpoint ratio in the progressive ratio test. **a** After completing outcome devaluation and contingency degradation tests, Vehicle (*n* = 19) and CORT (*n* = 26) mice were trained for 3 sessions on a variable ratio 3 (VR3) schedule and then tested in progressive ratio in 3 sessions separated by 2 days, under a linearly increasing, X + 4 (1, 5, 9, X + 4) progressive ratio schedule. **b** Chronic CORT reduced total active lever presses compared to Vehicle in all 3 sessions (*p* *<* 0.001). **c** Chronic CORT similarly reduced last ratio reached in all 3 sessions (*p* *<* 0.001). Values plotted are mean ± SEM. *p *<* 0.001. Taken together, chronic CORT reduces motivation to expend effort for food pellet reinforcers by reducing lever presses and breakpoint in three progressive ratio tests.

We next assessed the effect of CORT administration on three progressive ratio tests in the cohort previously exposed to the extended training to test if chronic CORT impairs motivation to expend effort for a reinforcer (Fig. 4a). A two-way repeated measures ANOVA revealed a main effect of CORT administration on active lever presses (*F*(1, 43) = 65.77, *p* *<* 0.001), and a main effect of day on active lever presses (*F*(2, 86) = 8.08, *p* *<* 0.001), but a non-significant interaction (*F*(2, 86) = 0.96, *p* *=* 0.387) (Fig. 4b; Supplemental Fig. 3a). Planned comparisons indicate that CORT reduced active lever presses compared to Vehicle in all three progressive ratio sessions (*p* < 0.001). For the three progressive ratio sessions, a two-way repeated measures ANOVA revealed a main effect of CORT administration on last ratio reached (*F*(1, 43) = 56.80, *p* *<* 0.001), and a main effect of day on last ratio reached (*F*(2, 86) = 4.98, *p* *=* 0.009), but a non-significant interaction (*F*(2, 86) = 0.41, *p* *=* 0.668) (Fig. 4c; Supplemental Fig. 3b). Therefore, chronic CORT treatment reduces motivation to lever press on a progressively increasing requirement, as CORT decreased breakpoint and total active lever presses.

### Probabilistic reversal learning

**Fig. 5:**
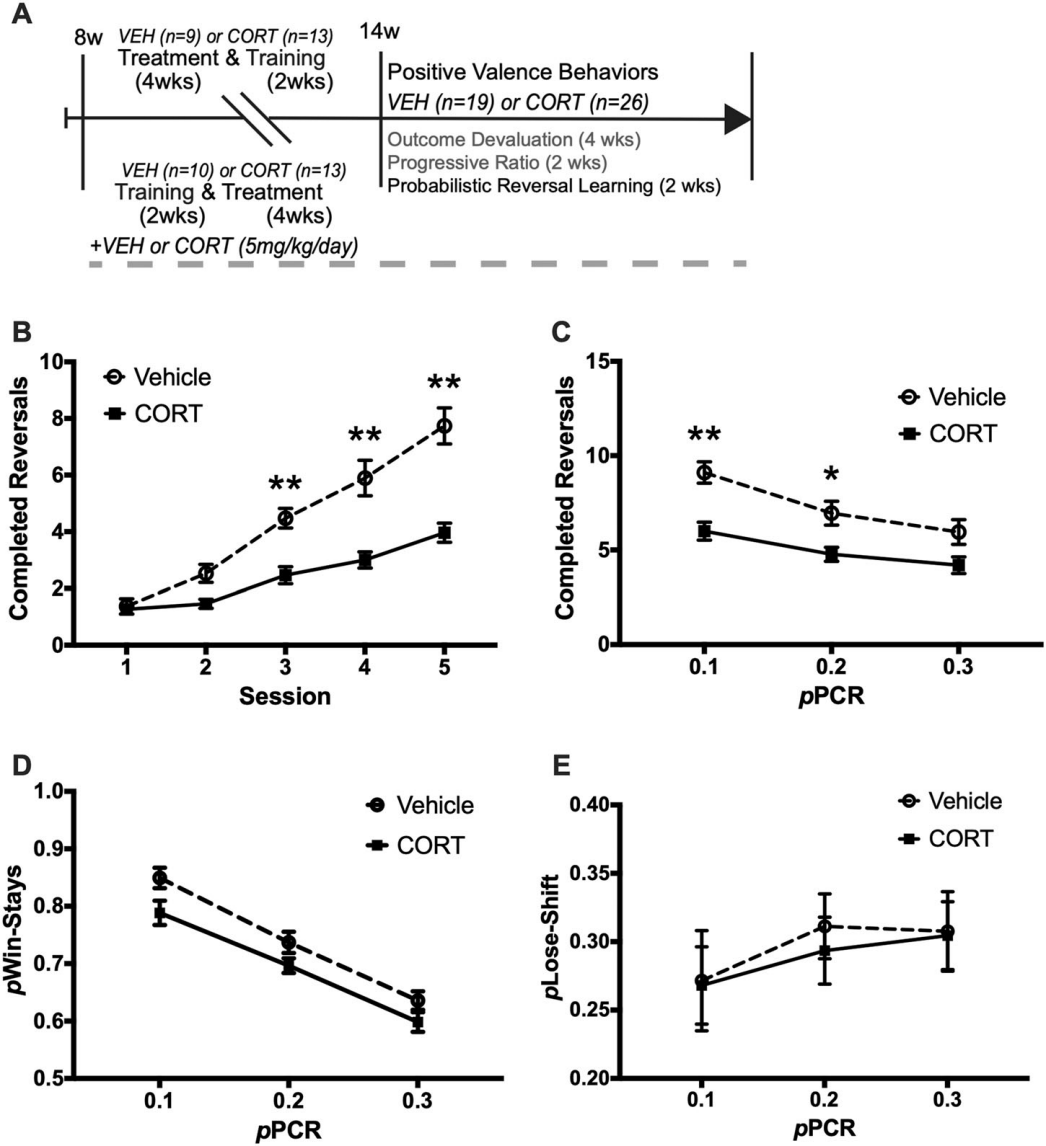
Chronic CORT impairs completed reversals in reversal learning and probabilistic reversal learning. **a** Vehicle (*n* = 19), and CORT (*n* = 26) mice were trained for 5 days on reversal learning, followed by three separate probabilistic reversal learning (PRL) test sessions, where the probability of punishment for a correct response (pPCR), was 0.1, 0.2, and 0.3, counterbalanced between mice. **b** Chronic CORT reduced completed reversals compared to Vehicle on reversal learning training days 3, 4, and 5 (*p* *<* 0.001). **c** Chronic CORT also reduced completed reversals in the 0.1 (*p* *<* 0.001) and 0.2 (*p* *=* 0.01) pPCR PRL test sessions, but not in the 0.3 pPCR PRL session (*p* *=* 0.38). **d** Chronic CORT trended to reduce the probability of making a win-stay in the 0.1 pPCR PRL session. **e** CORT has no effect on the probability of making a lose-shift in any of the PRL sessions. Values plotted are mean ± SEM. *p *<* 0.05, **p *<* 0.001. Taken together, chronic CORT impairs cognitive flexibility in both reversal learning and PRL sessions by reducing competed reversals. While non-significant, CORT also trends to reduce the probability of making a win-stay in the 0.1 pPCR PRL session (*p* = 0.052).

Lastly, we assessed the effects of CORT administration on probabilistic reversal learning in the cohort previously exposed to the extended training outcome devaluation and the progressive ratio (Fig. 5a). A two-way repeated measures ANOVA for completed reversals during the five days of reversal learning training revealed a main effect of CORT administration (*F*(1, 43) = 39.2, *p* *<* 0.001), a main effect of training day (*F*(4, 172) = 69.76, *p* *<* 0.001), and a significant interaction (*F*(4, 172) = 10.97, *p* *<* 0.001) (Fig. 5b; Supplemental Fig. 3c). Bonferroni’s multiple comparisons test indicated that CORT reduced completed reversals compared to Vehicle on training days 3, 4, and 5 (*p* *<* 0.001). Across the three sessions of probabilistic reversal learning, there were main effects of CORT administration (*F*(1, 43) = 26.37, *p* *<* 0.001), and probability of punishment for a correct response (pPCR) (*F*(2, 86) = 13.14, *p* *<* 0.001), on completed reversals, and a non-significant interaction *(F*(2, 86) = 0.97, *p* *<* 0.38 (Fig. 5c; Supplemental Fig. 3d). Planned comparisons indicated CORT reduced completed reversals when pPCR was 0.1 (*p* *<* 0.001), and 0.2 (*p* *=* 0.01), but not when pPCR was 0.3 (*p* *=* 0.054). Across the 3 days of probabilistic reversal learning, there was a main effect of pPCR on the probability of making a Win-Stay (*F*(2, 86) = 67.43, *p* *<* 0.001), and a main effect of CORT administration (*F*(1, 43) = 9.054, *p* = 0.004), but the interaction between factors was non-significant (*F*(2, 86) = 0.27, *p* *=* 0.77) (Fig. 5d; Supplemental Fig. 3e). While non-significant, planned comparisons indicated CORT trended to reduce the probability of making a Win-Stay when pPCR was 0.1 (*p* *=* 0.052). Across the three days of probabilistic reversal learning, there were no main effects of pPCR (*F*(2, 86) = 1.147, *p* *=* 0.322) or treatment *F*(1, 43) = 0.1, *p* = 0.754) on probability of making a lose-shift (Fig. 5e; Supplemental Fig. 3f). These data demonstrate that CORT impairs cognitive flexibility in both reversal learning and probabilistic reversal learning tasks, as CORT mice failed to complete as many reversals as Vehicle mice during training, and in all three PRL sessions. Further, CORT may impact positive feedback sensitivity, as CORT trended to reduce win-stays when pPCR was 0.1.

## Discussion

Similar to previous reports^1^, chronic CORT induced a robust anxiety-associated approach-avoidance negative valence behavioral phenotype in both the OF and NSF, while no effect was found in the FST, a test sensitive to antidepressant treatment that is not affected by chronic CORT^1^. CORT increased avoidance behaviors in the OF and increased latency to consume a pellet in the NSF, approach-avoidance conflict tasks which primarily assess innate anxiety amid the conflict between exploration and aversive environments such as the center of the OF, open arms of an EPM, or while food-deprived in the brightly-lit, novel NSF chamber^19–21^.

Here we show that chronic CORT administration also had several effects on instrumental behaviors including causing impairments in instrumental acquisition and limited-training satiety-specific outcome devaluation. CORT administration impaired motivation to expend effort for reinforcer pellets in progressive ratio, as lever pressing and total reinforcers earned were reduced. CORT administration also impaired performance in reversal learning and the PRL tests by reducing total number of completed reversals. Taken together, these data suggest that chronic CORT administration induces both an anxiety-related negative affective state and blunts instrumental reward processing. Our chronic CORT administration paradigm utilizes the lowest dose (5 mg/kg/day) possible that consistently induces negative valence behaviors^1^. Our data here suggests that this same administration paradigm is also useful for studying the effects of chronic stress on translationally relevant instrumental behaviors. Since disorders such as depression can involve both a gain of negative affect and a loss of positive affect in humans, the usage of both anxiety-related approach-avoidance behaviors associated with negative valence and instrumental behaviors associated with positive valence in rodents provides a complete picture of the effects of chronic stress paradigms on behavior.

Acquisition involves the formation of an action-outcome (A-O) contingency, as lever pressing leads to reward presentation^22^. Here we show chronic CORT delays acquisition, similar to previous findings^4^. This suggests that this chronic stressor is impairing the formation of the A-O contingency, as CORT-administered mice take longer training to reach the acquisition criterion. Previous research using chronic CORT administered after instrumental acquisition has found that progressive ratio and sucrose preference are reduced, indicating impaired reward motivation and anhedonia^2^. Our results support this finding, as mice administered chronic CORT had a lower breakpoint and total active lever presses in progressive ratio.

Devaluing the instrumental response via outcome devaluation or contingency degradation tests if behavior is controlled by a goal-directed (A-O) or stimulus-response (S-R), habitual mechanism. While non-stressed mice reduce responding for devalued food pellets (via contingency degradation), chronic CORT-treated mice maintained their level of responding, indicating an S-R, habitual mechanism underlying this pattern of responding. Thus, chronic CORT reduces sensitivity to a devalued outcome in satiety-specific outcome devaluation. Chronic social defeat stress also reduces A-O decision-making, therefore reducing responding for a devalued reinforcer using a satiety-specific outcome devaluation^22^. Therefore, given our data here, chronic stress paradigms such as CORT administration and social defeat stress originally designed for and validated using anxiety-related approach-avoidance behaviors associated with negative valence can also be used to determine the effects of chronic stress on instrumental behaviors associated with positive valence.

To better understand whether there is impaired associative learning, we calculated a learning index (correct lever presses during acquisition training divided by total number of trials in each session)^23,24^, whereby we determined that Vehicle and CORT-administered mice receiving treatment throughout training reach a similar learning index prior to positive valence behavioral testing. Thus, these mice do not progress to any stage of training for each of these behavioral tests until reaching a criterion (defined as consecutive sessions with ≥30 lever presses on both response levers), to ensure that they have fully learned the task. In addition, CORT administration did not affect associative learning in a one-trial contextual fear conditioning paradigm. Therefore, while these data suggest that CORT does not impair associative learning, we cannot rule out this possibility out entirely.

Other studies have utilized subchronic and/or higher doses of CORT to assess effects on positive valence behaviors in rats and mice and have reported mixed results. One study found that subchronic CORT (at a dose ~60% higher than we used) impairs instrumental acquisition^4^, while another found that subchronic CORT (at a dose similar to what we used) had no effect on instrumental acquisition^15^. Both of these studies utilized nose poke apertures for mice during the acquisition. Our chronic CORT administration protocol, which uses a dose well-validated for negative valence behaviors (5 mg/kg/day)^1^ resulted in impaired instrumental acquisition using lever presses during the acquisition. Furthermore, subchronic CORT (at a dose similar to what we used) impaired both lithium chloride-paired outcome devaluation and also contingency degradation in mice utilizing nose poke apertures^15^. We found that chronic CORT impaired satiety-specific outcome devaluation in mice utilizing lever presses. However, we did not see a significant effect of CORT administration on contingency degradation. Extended-training mice showed significant effects of degradation, as lever pressing was reduced in the extinction test session, regardless of vehicle or chronic CORT treatment, and there was no effect of degradation in the limited-training cohort, regardless of vehicle or chronic CORT treatment. Subchronic CORT (using doses either 40% higher or 540% higher than we used) reduced nose poke responding in a progressive ratio task^2,25^. Finally, our chronic CORT administration paradigm lasted the entirety of behavioral testing (8–12 weeks), whereas others have used 2–4 weeks of treatment followed by weaning mice off the CORT. But, this paradigm allows behavioral testing for only about 4 weeks after weaning off CORT^2,25^. As weaning off the CORT limits the time-frame in which significant effects are found, our paradigm allows for more long-term investigation of the behavioral impact of chronic CORT administration. Importantly, to our knowledge the data presented here are also the first to assess the effects of CORT administration on probabilistic reversal learning.

Probabilistic reversal learning assesses behavioral flexibility, and the sensitivity to both positive and negative feedback^17,18^. In a reversal learning procedure rodents respond to a correct lever for a reward pellet on eight consecutive trials. Then, the rewarded lever switches sides, and the rodent must respond on the other lever to receive a reward. This contingency switches throughout the reversal learning session, and the number of completed reversals is considered a measure of cognitive flexibility. In the probabilistic versions of this test correct responses are not reinforced, while incorrect responses are reinforced at a determined probability. This provides positive and negative feedback, which are assessed with win-stays and lose-shifts during the task. We found that chronic CORT reduced completed reversals in both training and during probabilistic versions of the test, and that win-stays trended to be reduced in CORT mice when pPCR was 0.1. This novel finding further demonstrates that the CORT paradigm produces a robust blunting of positive valence throughout several behavioral tests.

Correspondence between human and rodent measures of reward learning, such as in the PRL task, is especially high^8,26^. The human versions of PRL^26^ do not rely on self-reporting and the rodent versions of PRL were actually developed based on the parameters used in humans^17,18^. In the human version of the PRL task subjects must learn to alter responding to a reversed contingency and ignore both positive and negative misleading feedback to maximize the probability of reward^27^. Humans must visually discriminate between stimuli on a computer screen, which can be abstract patterns or lines of similar length^28^. While they are rewarded for correct choices, the stimuli reverse just like in the rodent version of this task. Negative feedback occurs similarly as the rodent version, with the same pPCR of 0.1, 0.2, and 0.3. In agreement with previous findings in rodents manipulating central serotonin levels^17,18^, depressed patients show greater focus on negative feedback in the PRL test^26^. While previous rodent work has assessed the effects of various antidepressant treatments on PRL, this study provides the first evidence that a chronic stressor such as CORT leads to similar effects in PRL as to what is observed in depressed patients.

Mood disorders are mediated by a complex neural circuitry involving interconnected regions including the nucleus accumbens, medial prefrontal cortex, amygdala, ventral hippocampus, and ventral tegmental area^29^. Dysfunction in distinct circuits likely mediate the different symptoms observed in mood disorders. While significant effort has helped decipher the neural circuitry underlying negative valence behaviors, especially those associated with anxiety^19^, less is known about the circuitry mediating positive valence behaviors. Therefore, future studies will need to also focus on deciphering the neural circuitry of positive valence and the effects of chronic stress on this circuitry.

While others have used the same cohort in successive behavior tests^4^, and we re-trained all mice prior to each test session, one limitation of our experiments is that we used the same cohort of mice for multiple operant behavioral tests. Therefore, we cannot rule out potential effects of previous instrumental test exposures. Future studies should assess the effects of antidepressant treatment on these chronic CORT-induced negative and positive valence behavioral effects. While chronic CORT is not a useful model in female mice^30^, future work should also examine the effect of chronic stressors on positive valence in females, as depression is more prevalent in women^31^, and preclinical depression research in female rodents is still lacking.

## Supplementary information

Supplemental Figure Legends

Supplemental Figure 1

Supplemental Figure 2

Supplemental Figure 3

## References

[1] David, D. J. et al. Neurogenesis-dependent and-independent effects of fluoxetine in an animal model of anxiety/depression. *Neuron* **62**, 479–493 (2009). 10.1016/j.neuron.2009.04.017 PMC2759281 19477151

[2] Gourley, S. L., Kiraly, D. D., Howell, J. L., Olausson, P. & Taylor, J. R. Acute hippocampal brain-derived neurotrophic factor restores motivational and forced swim performance after corticosterone. *Biol. Psychiatry* **64**, 884–890 (2008). 10.1016/j.biopsych.2008.06.016 PMC2633780 18675955

[3] Gourley, S. L. & Taylor, J. R. Recapitulation and reversal of a persistent depression‐like syndrome in rodents. *Curr. Protoc. Neurosci*. **49**, 9.32.1–9.32.11 (2009). 10.1002/0471142301.ns0932s49 PMC2774936 19802817

[4] Olausson, P., Kiraly, D. D., Gourley, S. L. & Taylor, J. R. Persistent effects of prior chronic exposure to corticosterone on reward-related learning and motivation in rodents. *Psychopharmacology* **225**, 569–577 (2013). 10.1007/s00213-012-2844-4 PMC3546199 22983097

[5] School, H. M. National Comorbidity Survey (NCS). (2007).

[6] Ahrnsbrak, R., Bose, J., Hedden, S. L., Lipari, R. N. & Park-Lee, E. Key substance use and mental health indicators in the United States: Results from the 2016 National Survey on Drug Use and Health (HHS Publication No. SMA 17-5044, NSDUH Series H-52). (2017).

[7] Kessler R (2003). Epidemiology of women and depression. Journal of Affective Disorders.

[8] Der-Avakian, A., Barnes, S. A., Markou, A. & Pizzagalli, D. A. in *Translational Neuropsychopharmacology* p. 231–262 (Springer, 2015).

[9] Vrieze Elske, Pizzagalli Diego A., Demyttenaere Koen, Hompes Titia, Sienaert Pascal, de Boer Peter, Schmidt Mark, Claes Stephan (2013). Reduced Reward Learning Predicts Outcome in Major Depressive Disorder. Biological Psychiatry.

[10] Schwabe L, Wolf OT (2010). Socially evaluated cold pressor stress after instrumental learning favors habits over goal-directed action. Psychoneuroendocrinology.

[11] Roane HS, Lerman DC, Vorndran CM (2001). Assessing reinforcers under progressive schedule requirements. J. Appl. Behav. Anal..

[12] Frank MJ, Seeberger LC, O'Reilly R, By C (2004). By carrot or by stick: cognitive reinforcement learning in parkinsonism. Science.

[13] Pechtel P, Pizzagalli DA (2013). Disrupted reinforcement learning and maladaptive behavior in women with a history of childhood sexual abuse: a high-density event-related potential study. JAMA Psychiatry.

[14] Gotlib IH, Joormann J, Minor KL, Hallmayer J (2008). HPA axis reactivity: a mechanism underlying the associations among 5-HTTLPR, stress, and depression. Biol. Psychiatry.

[15] Gourley S. L., Swanson A. M., Jacobs A. M., Howell J. L., Mo M., DiLeone R. J., Koleske A. J., Taylor J. R. (2012). Action control is mediated by prefrontal BDNF and glucocorticoid receptor binding. Proceedings of the National Academy of Sciences.

[16] Zimmermann, K. S., Yamin, J. A., Rainnie, D. G., Ressler, K. J. & Gourley, S. L. Connections of the mouse orbitofrontal cortex and regulation of goal-directed action selection by brain-derived neurotrophic factor. *Biol Psychiatry* **81**, 366–377 (2017). 10.1016/j.biopsych.2015.10.026 PMC4871791 26786312

[17] Bari Andrea, Theobald David E, Caprioli Daniele, Mar Adam C, Aidoo-Micah Alex, Dalley Jeffrey W, Robbins Trevor W (2010). Serotonin Modulates Sensitivity to Reward and Negative Feedback in a Probabilistic Reversal Learning Task in Rats. Neuropsychopharmacology.

[18] Ineichen Christian, Sigrist Hannes, Spinelli Simona, Lesch Klaus-Peter, Sautter Eva, Seifritz Erich, Pryce Christopher R. (2012). Establishing a probabilistic reversal learning test in mice: Evidence for the processes mediating reward-stay and punishment-shift behaviour and for their modulation by serotonin. Neuropharmacology.

[19] Calhoon, G. G. & Tye, K. M. Resolving the neural circuits of anxiety. *Nat. Neurosci.* **18**, 1394–1404 (2015). 10.1038/nn.4101 PMC7575249 26404714

[20] Borsini F, Podhorna J, Marazziti D (2002). Do animal models of anxiety predict anxiolytic-like effects of antidepressants?. Psychopharmacology.

[21] Pellow S, Chopin P, File SE, Briley M (1985). Validation of open:closed arm entries in an elevated plus-maze as a measure of anxiety in the rat. J. Neurosci. Methods.

[22] Dias-Ferreira E., Sousa J. C., Melo I., Morgado P., Mesquita A. R., Cerqueira J. J., Costa R. M., Sousa N. (2009). Chronic Stress Causes Frontostriatal Reorganization and Affects Decision-Making. Science.

[23] Garren, M. V., Sexauer, S. B. & Page, T. L. Effect of circadian phase on memory acquisition and recall: operant conditioning vs. classical conditioning. *PLoS ONE* **8**, e58693 (2013). 10.1371/journal.pone.0058693 PMC3606338 23533587

[24] Brush F. R., Brush E. S., Solomon R. L. (1955). Traumatic avoidance learning: the effects of CS-US interval with a delayed-conditioning procedure. Journal of Comparative and Physiological Psychology.

[25] Gourley Shannon L., Wu Florence J., Kiraly Drew D., Ploski Jonathan E., Kedves Alexia T., Duman Ronald S., Taylor Jane R. (2008). Regionally Specific Regulation of ERK MAP Kinase in a Model of Antidepressant-Sensitive Chronic Depression. Biological Psychiatry.

[26] Taylor Tavares Joana V., Clark Luke, Furey Maura L., Williams Guy B., Sahakian Barbara J., Drevets Wayne C. (2008). Neural basis of abnormal response to negative feedback in unmedicated mood disorders. NeuroImage.

[27] Cools, R., Clark, L., Owen, A. M. & Robbins, T. W. Defining the neural mechanisms of probabilistic reversal learning using event-related functional magnetic resonance imaging. *J. Neurosci.* **22**, 4563–4567 (2002). 10.1523/JNEUROSCI.22-11-04563.2002 PMC6758810 12040063

[28] Evers Elizabeth A T, Cools Roshan, Clark Luke, van der Veen Frederik M, Jolles Jelle, Sahakian Barbara J, Robbins Trevor W (2005). Serotonergic Modulation of Prefrontal Cortex during Negative Feedback in Probabilistic Reversal Learning. Neuropsychopharmacology.

[29] Nestler, E. J. & Carlezon Jr, W. A. The mesolimbic dopamine reward circuit in depression. *Biol. Psychiatry* **59**, 1151–1159 (2006). 10.1016/j.biopsych.2005.09.018 16566899

[30] Mekiri, M., Gardier, A. M., David, D. J. & Guilloux, J.-P. Chronic corticosterone administration effects on behavioral emotionality in female c57bl6 mice. *Exp. Clin. Psychopharmacol.* **25**, 94–104 (2017). 10.1037/pha0000112 28287792

[31] Albert Paul (2015). Why is depression more prevalent in women?. Journal of Psychiatry & Neuroscience.

