# Supplementary material for: Chronic corticosterone administration induces negative valence and impairs positive valence behaviors in mice
Source: Transl Psychiatry. 2019 Dec 10;9:337. doi: 10.1038/s41398-019-0674-4 (PMC6904464; doi:10.1038/s41398-019-0674-4)
Supplement: Supplementary file 1 — Supplemental Figure Legends [file 41398_2019_674_MOESM1_ESM.docx]

**Supplemental Figure 1.**

(A) In the novelty-suppressed feeding assay, Vehicle (n=10) and CORT-administered (n=10) mice have similar latency to eat in the home cage. (B) In an outcome devaluation 1-hour satiety home cage feeding test, reinforcer pellet consumption (mg) per body weight (g) does not differ between Vehicle (n=10) and CORT-administered (n=10) mice.

**Supplemental Figure 2.**

A learning index was calculated (correct responses divided by total trials per session) to determine if the differences seen in instrumental acquisition can be attributed to impaired associative learning. (A) Displays the progressive increase in learning index across Acquisition for all four groups (Vehicle after training; Vehicle throughout training; CORT after training; CORT throughout training). (B) Learning index is similar for mice administered Vehicle and CORT throughout training in Session 6. (C) Freezing during a contextual fear conditioning retrieval session does not differ between Vehicle and CORT-administered mice, suggesting no deficit in associative learning caused by chronic CORT.

**Supplemental Figure 3.**

Individual data from graphs presented in Figures 4 and 5. (A) Active lever presses and (B) Last Ratio Reached in the 3 Progressive Ratio sessions for Vehicle and CORT-administered mice. (C) Completed reversals during the 5 training sessions of Reversal Learning for Vehicle and CORT mice. (D) Completed reversals, (E) *p*Win-Stays, and (F) *p*Lose-Shifts for Vehicle and CORT-administered mice during the 3 Probabilistic Reversal Learning test sessions.
