# Supplementary figures and images for: Chronic corticosterone administration induces negative valence and impairs positive valence behaviors in mice
Source: Transl Psychiatry. 2019 Dec 10;9:337. doi: 10.1038/s41398-019-0674-4 (PMC6904464; doi:10.1038/s41398-019-0674-4)

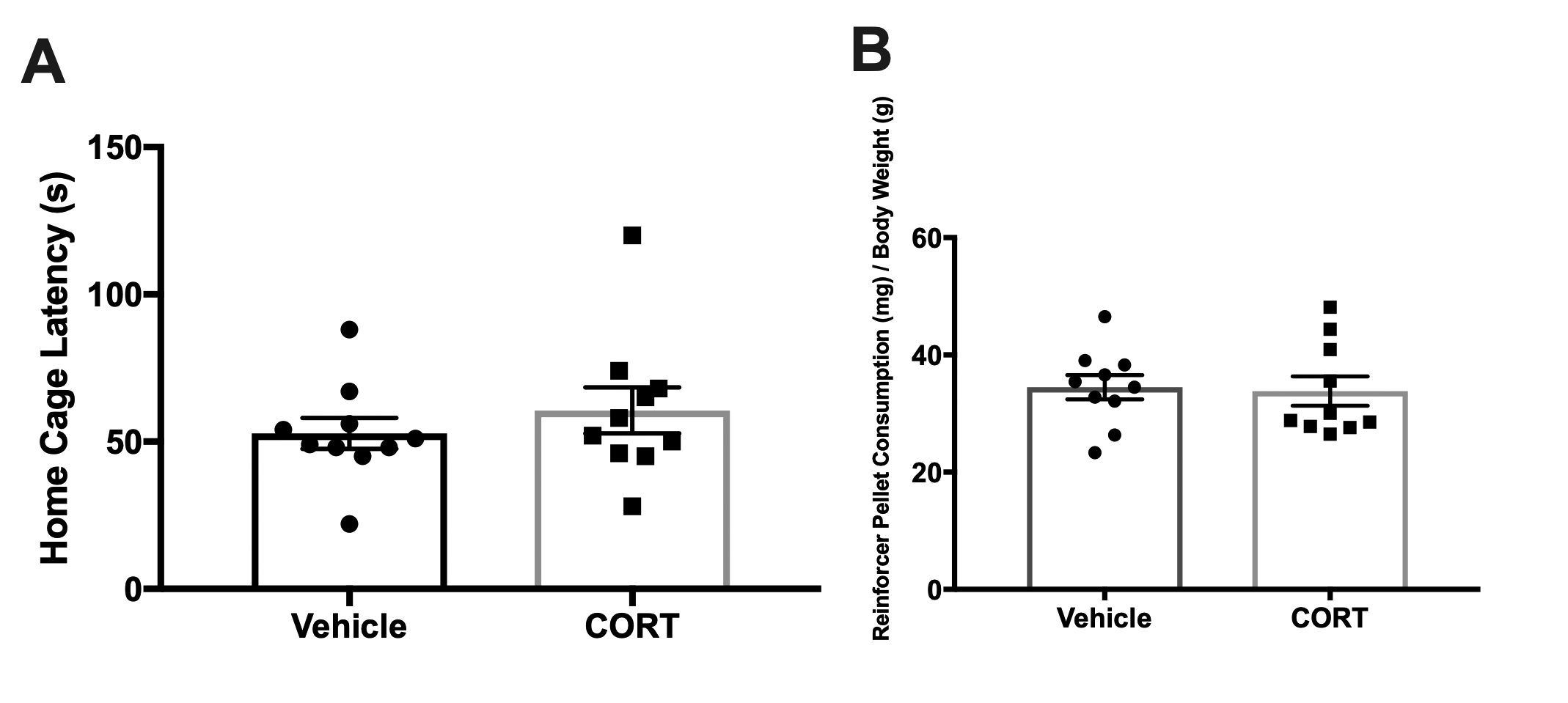

Supplement: Supplementary file 2 — Supplemental Figure 1 [file 41398_2019_674_MOESM2_ESM.tif]

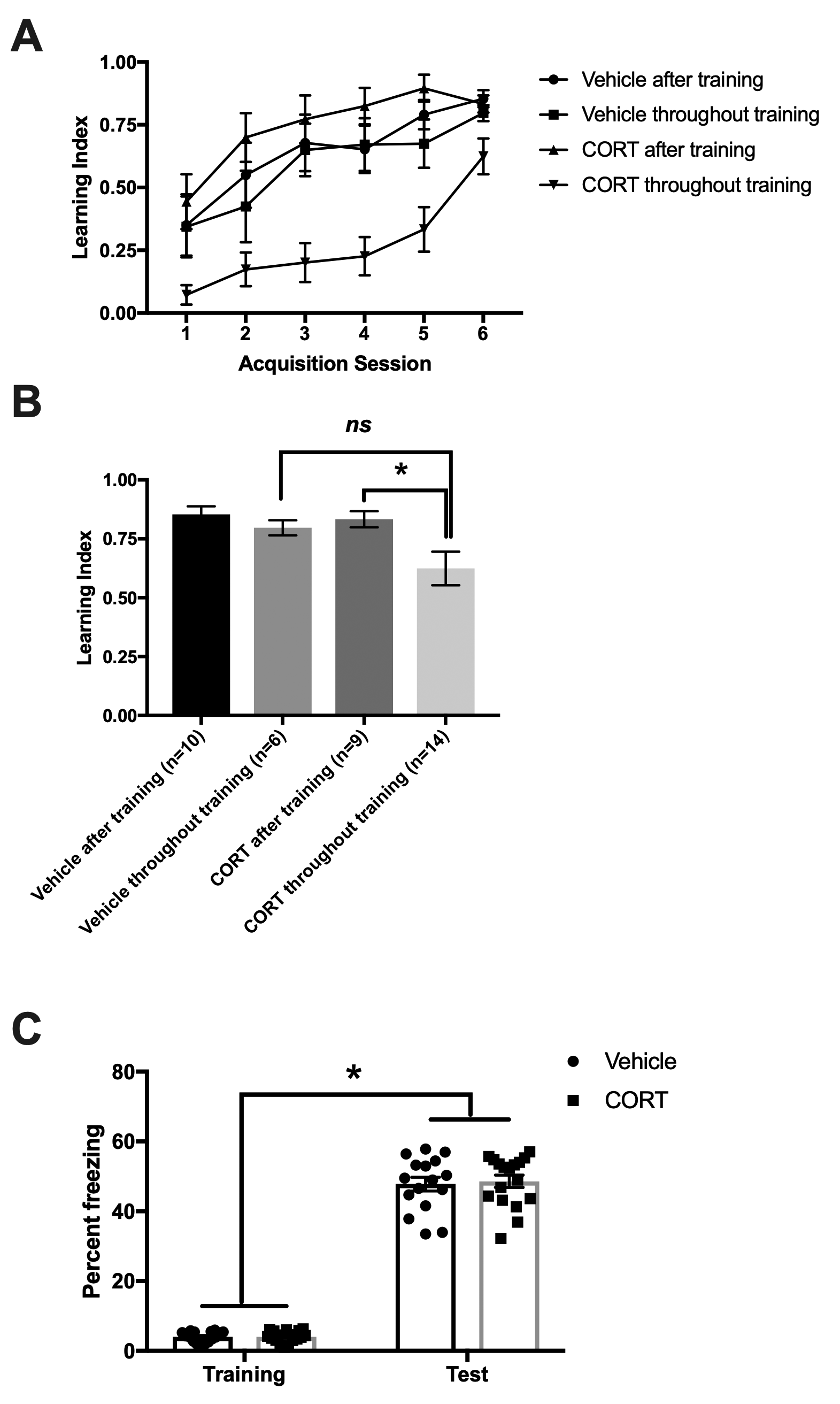

Supplement: Supplementary file 3 — Supplemental Figure 2 [file 41398_2019_674_MOESM3_ESM.tif]

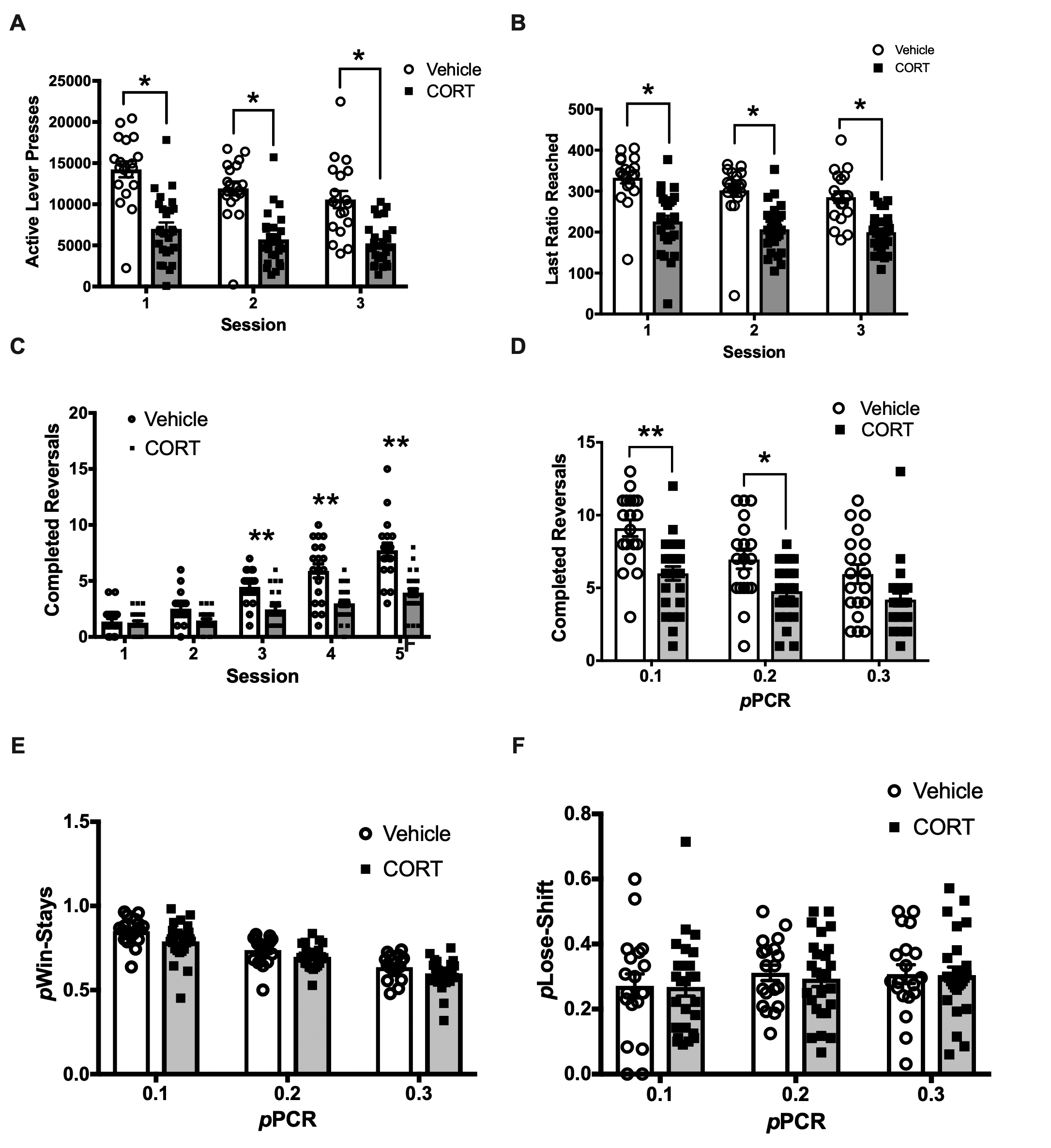

Supplement: Supplementary file 4 — Supplemental Figure 3 [file 41398_2019_674_MOESM4_ESM.tif]
